# The Structure of EAP-Groups and Self-Autopermutable Subgroups

**DOI:** 10.1155/2014/850526

**Published:** 2014-12-11

**Authors:** Shima Housieni, Mohammad Reza Rajabzadeh Moghaddam

**Affiliations:** ^1^Department of Mathematics, Islamic Azad University, Mashhad Branch, Mashhad 9187147578, Iran; ^2^Department of Mathematics, Khayyam University, Mashhad 9189747178, Iran; ^3^Centre of Excellence in Analysis on Algebraic Structures, Ferdowsi University of Mashhad, P.O. Box 1159-91775, Iran

## Abstract

A subgroup *H* of a given group *G* is said to be *autopermutable*, if *HH* ^*α*^ = *H* ^*α*^ *H* for all *α* ∈ Aut(*G*). We also call *H* a *self-autopermutable subgroup* of *G*, when *HH* ^*α*^ = *H* ^*α*^ *H* implies that *H* ^*α*^ = *H*. Moreover, *G* is said to be *EAP-group*, if every subgroup of *G* is *autopermutable*. One notes that if *α* runs over the inner automorphisms of the group, one obtains the notions of conjugate-permutability, self-conjugate-permutability, and *ECP-groups*, which were studied by Foguel in 1997, Li and Meng in 2007, and Xu and Zhang in 2005, respectively. In the present paper, we determine the structure of a finite EAP-group when its centre is of index 4 in *G*. We also show that self-autopermutability and characteristic properties are equivalent for nilpotent groups.

## 1. Introduction

Let *H* be a subgroup of a given group *G*. Then we call *H* to be autopermutable, if *HH* ^*α*^ = *H* ^*α*^ *H* for all *α* ∈ Aut(*G*). The subgroup *H* is said to be self-autopermutable, if *HH* ^*α*^ = *H* ^*α*^ *H* implies that *H* ^*α*^ = *H*. Moreover, we call the group *G* to be an EAP-group if every subgroup of *G* is autopermutable. Clearly, if *α* runs over the inner automorphisms of the group, we obtain the notions of conjugate-permutability [1], self-conjugate-permutability [2], and ECP-groups [3], respectively. One notes that the subgroup *H* = 〈*b*〉 of the Dihedral group *D*_8_ = 〈*a*, *b* : *a* ^4^ = *b* ^2^ = 1, *a* ^*b*^ = *a* ^−1^〉 is conjugate-permutable, which is not autopermutable. To see this, consider the automorphism *α* which sends *a* and *b* into *a* and *ab*, respectively. Clearly *HH* ^*α*^ is not a subgroup of *G*, which means that *HH* ^*α*^ ≠ *H* ^*α*^ *H*. It is easily seen that similar examples can be obtained by taking a direct product of *D*_8_ with any other group. Also, every noncharacteristic normal subgroup of a given group is an example for a self-conjugate-permutable subgroup which is not self-autopermutable. Moreover, *D*_8_ is an ECP-group, which is not an EAP-group.

In the present paper, we determine the structure of a finite EAP-group, when its centre is of index 4. We also prove that self-autopermutability and characteristic properties are equivalent in nilpotent groups.

## 2. Finite EAP-Groups

In this section, we determine the structure of finite EAP-groups, when their centres are of index 4. In fact we prove the following theorem.

**Theorem 1 .**

Let *G* be a finite group with the centre of index 4. Then *G* is an EAP-group if and only if the Sylow 2-subgroup of *G* is one of the following forms:

- *Q*_8_;
- 〈*a*, *b*∣*a* ^2^*n*+1^^ = *b* ^2^ = 1, *a* ^*b*^ = *a* ^2^*n*^+1^〉, *n* ≥ 3;
- *Z*_2_ × *Q*_8_;
- 〈*a*, *b*, *c*, *d*∣*a* ^2^ = *b* ^2^ = *c* ^4^ = *d* ^4^ = 1, *b* ∈ *Z*(*G*), [*a*, *c*] = [*a*, *d*] = *c* ^3^ *d*, [*c*, *d*] = 1〉;
- 〈*a*, *b*, *c*, *d*, *e*∣*a* ^2^ = *b* ^2^ = *c* ^2^ = *d* ^2^ = *e* ^4^ = 1, *a*, *b*, *c* ∈ *Z*(*G*), [*d*, *e*] = *b*〉;
- 〈*a*, *b*, *c*, *d*, *e*∣*a* ^2^ = *b* ^2^ = *c* ^2^ = *d* ^4^ = *e* ^4^ = 1, *a*, *b*, *c* ∈ *Z*(*G*), [*d*, *e*] = *a*〉.

We remind that a nonabelian group is said to be Hamiltonian, if all of its subgroups are normal. The following result gives our claim, when *G* is a 2-group with cyclic centre of index 4.

**Theorem 2 .**

Let *G* be a finite 2-group with cyclic centre of index 4. Then *G* is an EAP-group if and only if *G*≅*Q*_8_ or 〈*a*, *b*∣*a* ^2^*n*+1^^ = *b* ^2^ = 1, *a* ^*b*^ = *a* ^2^*n*^+1^〉, for all *n* ≥ 3.

**Proof**

Consider the group *G* to be *Q*_8_. Since *Q*_8_ is Hamiltonian group, the result follows easily. Now assume *G* = 〈*a*, *b*∣*a* ^2^*n*+1^^ = *b* ^2^ = 1, *a* ^*b*^ = *a* ^2^*n*^+1^〉, *n* ≥ 3. One can easily check that *G* contains exactly three proper subgroups of orders 2^*i*^, for 1 ≤ *i* ≤ *n* + 1. We also observe that the subgroups of orders 2 are autopermutable and as the subgroups of orders 2^*n*+1^ are normal, they are also autopermutable. Now, one can check that there are exactly two cyclic and one noncyclic subgroups of orders 2^*i*^, 2 ≤ *i* ≤ *n*, so that one of the cyclic subgroups is central and hence all the subgroups of *G* satisfy the required property.

Conversely, assume that *G* is an EAP-group, *Z*(*G*) = 〈*x*∣*x* ^2^*n*^^ = 1〉, *G*/*Z*(*G*) = {*Z*(*G*), *aZ*(*G*), *bZ*(*G*), *ab* *Z*(*G*)}, where *a* ^2^, *b* ^2^ ∈ *Z*(*G*) and so |*a*|, |*b*| ≤ 2^*n*+1^. In case *n* = 1, then the group *G* is either *D*_8_ or *Q*_8_. As explained before, *D*_8_ cannot be an EAP-group and hence *G*≅*Q*_8_. Now suppose *n* > 1 and the elements *a* and *b* are both of order 2. Then every element *y* ∈ *G* has the following form (as *G* is nilpotent of class 2):

(1) $$\begin{array}{c} y=a^{i}b^{j}x^{k},\;\text{where}\;\;0\leq i,j<2,\;\;0\leq k<2^{n}. \end{array}$$

Clearly, the map *α* given by *α*(*y*) = *b* ^*i*^ *a* ^*j*^ *x* ^*k*^ is an automorphism of *G*, which sends *a* into *b*. Thus *HH* ^*α*^ ≠ *H* ^*α*^ *H* for the subgroup *H* = 〈*b*〉, which contradicts the assumption. Now, if |*a* | , |*b* | <2^*n*+1^ we may replace *a* and *b* by the elements *ax* ^*i*^ and *bx* ^*j*^, both of which are of order 2. This reduces to the previous case. Therefore we must have *a* or *b* of order 2^*n*+1^. Then *G* has a cyclic subgroup of order 2^*n*+1^ and so *G* is of order 2^*n*+2^  (*n* > 1) with the centre of index 4. Hence, by [4, 5.3.4], the group *G* has the following presentation:

(2) $$\begin{array}{c} G=\langle a,b\;|\;a^{2^{n+1}}=b^{2}=1,a^{b}=a_{}^{2^{n}+1}\rangle ,\;n\geq 3. \end{array}$$

This is an EAP-group and so the proof is completed.

*The following result considers the case when G* *is a 2-group with noncyclic centre of index 4.*

**Theorem 3 .**

Let *G* be a finite 2-group with noncyclic centre of index 4. Then *G* is an EAP-group if and only if G is one of the following forms:

- *G* = *Z*_2_ × *Q*_8_;
- *G* = 〈*a*, *b*, *c*, *d*∣*a* ^2^ = *b* ^2^ = *c* ^4^ = *d* ^4^ = 1, *b* ∈ *Z*(*G*), [*a*, *c*] = [*a*, *d*] = *c* ^3^ *d*, [*c*, *d*] = 1〉;
- *G* = 〈*a*, *b*, *c*, *d*, *e*∣*a* ^2^ = *b* ^2^ = *c* ^2^ = *d* ^2^ = *e* ^4^ = 1, *a*, *b*, *c* ∈ *Z*(*G*), [*d*, *e*] = *b*〉;
- *G* = 〈*a*, *b*, *c*, *d*, *e*∣*a* ^2^ = *b* ^2^ = *c* ^2^ = *d* ^4^ = *e* ^4^ = 1, *a*, *b*, *c* ∈ *Z*(*G*), [*d*, *e*] = *a*〉.

**Proof**

The sufficient condition is obvious. We only need to prove the necessity condition. Let *G* be an EAP-group and *G*/*Z*(*G*) = {*Z*(*G*), *aZ*(*G*), *bZ*(*G*), *ab* *Z*(*G*)}, where *a* ^2^, *b* ^2^ ∈ *Z*(*G*). Assume that *Z*(*G*) is not an elementary abelian 2-group. Since *Z*(*G*) is the direct product of its cyclic subgroups, by the same argument as in Theorem 2, there are no EAP-groups in this case. Now, assume that *Z*(*G*) is an elementary abelian 2-group. Clearly *G* must be a group of order either 16 or 32. The structure of such groups is given as follows in [5]. If |*G*| = 16, then

- *G* = *Z*_2_ × *D*_8_;
- *G* = 〈*a*, *b*∣*a* ^4^ = *b* ^4^ = 1, *b* *ab* ^−1^ = *a* ^3^〉≅*Z*_4_⋊*Z*_4_;
- *G* = *Z*_2_ × *Q*_8_;
- *G* = 〈*a*, *b*, *c*, *d*∣*a* ^2^ = *b* ^2^ = *c* ^4^ = *d* ^4^ = 1, *b* ∈ *Z*(*G*), [*a*, *c*] = [*a*, *d*] = *c* ^3^ *d*, [*c*, *d*] = 1〉.

As *D*_8_ is not an EAP-group, hence the group of form (i) cannot be an EAP-group. For the group of form (ii) we can consider *H* = 〈*b*〉 and *α* ∈ Aut(*G*) which sends *a* and *b* into *a* and *ab*, respectively. Clearly, *HH* ^*α*^ ≠ *H* ^*α*^ *H* and hence *G* cannot be an EAP-group. Thus when |*a* | = |*b*|, then *G* is of the form given in either (iii) or (iv).

Assume |*G*| = 32. Then such groups in the list of small groups with elementary abelian centres of index 4 are only of the following forms:

- *G* = 〈*a*, *b*, *c*∣*a* ^4^ = *b* ^4^ = *c* ^2^ = 1, [*a*, *b*] = *c*, [*a*, *c*] = [*b*, *c*] = 1〉;
- *G* = 〈*a*, *b*, *c*, *d*, *e*∣*a* ^2^ = *b* ^2^ = *c* ^2^ = *d* ^4^ = *e* ^4^ = 1, *a*, *b*, *c* ∈ *Z*(*G*), [*d*, *e*] = *b*〉;
- *G* = 〈*a*, *b*, *c*, *d*, *e*∣*a* ^2^ = *b* ^2^ = *c* ^2^ = *d* ^2^ = *e* ^2^ = 1, *c*, *d* ∈ *Z*(*G*), [*a*, *e*] = [*b*, *e*] = *ab*, [*a*, *b*] = 1〉;
- *G* = 〈*a*, *b*, *c*, *d*, *e*∣*a* ^2^ = *b* ^2^ = *c* ^2^ = *d* ^2^ = *e* ^4^ = 1, *a*, *b*, *c* ∈ *Z*(*G*), [*d*, *e*] = *b*〉;
- *G* = 〈*a*, *b*, *c*, *d*, *e*∣*a* ^2^ = *b* ^2^ = *c* ^2^ = *d* ^4^ = *e* ^4^ = 1, *a*, *b*, *c* ∈ *Z*(*G*), [*d*, *e*] = *a*〉.

For the group of form (i) we may consider the cyclic subgroup *H* = 〈*b*〉 and *α* ∈ Aut(*G*), which sends *a*, *b*, and *c* into *a*, *ab*, and *c*, respectively. In case the group *G* is of form (ii), we consider *H* = 〈*e*〉 and *α* ∈ Aut(*G*) which sends *a*, *b*, *c*, *d*, and *e* into *a*, *ab*, *ac*, *be*, and *ed*, respectively. Also if the group is considered to be of form (iii), one may consider *H* = 〈*e*〉 and *α* ∈ Aut(*G*) which sends *a*, *b*, *c*, *d*, and *e* into *e*, *b*, *c*, *d*, and *a*, respectively. Now, one can easily check that in these cases *HH* ^*α*^ ≠ *H* ^*α*^ *H* and so *G* cannot be an EAP-group. Hence, when |*G* | = 32, then *G* is of either form (iv) or form (v). The proof is complete.

**Proof of Theorem 1.**

The necessity condition is obvious and Theorems 2 and 3 establish the result, when *G* is a 2-group. If *G* is not a 2-group, then we may write *G* = *S*_1_ × *S*_2_ ⋯ ×*S*_*k*_, in such a way that *S*_1_ is a Sylow 2-subgroup and *S*_*i*_ is an abelian Sylow *p*_*i*_-subgroup, where *p*_*i*_ is an odd prime number, for 2 ≤ *i* ≤ *k*. Clearly, Aut(*G*)≅Aut(*S*_1_) × Aut(*S*_2_)×⋯×Aut(*S*_*k*_) and for any subgroup *H* of *G*, *H*≅*H*_1_ × *H*_2_ × ⋯×*H*_*k*_, where *H*_*i*_ ≤ *S*_*i*_ for 1 ≤ *i* ≤ *k*. Thus *H* is an autopermutable subgroup of *G* if *H*_1_ is an autopermutable subgroup of *S*_1_. This completes the proof.

## 3. Self-Autopermutable Subgroups in Nilpotent Groups

We call a subgroup *H* of a given group *G* to be* weakly characteristic,* when *H* ^*α*^ ≤ *N*_*G*_(*H*) implies that *H* ^*α*^ = *H* for all *α* ∈ Aut(*G*). Also, given the subgroups *H* and *K*, then *H* satisfies the* subcharacteriser condition*, if *H*⊴*K* implies that *N*_Aut(*G*)_(*K*) ≤ *N*_Aut(*G*)_(*H*), where *N*_Aut(*G*)_(*K*) = {*α* ∈ Aut(*G*); *K* ^*α*^ = *K*} ≤ Aut(*G*). Clearly, if one considers the inner automorphisms of the group then* weakly normal* and* normaliser condition* properties are obtained.

The following result of [6] shows that self-conjugate-permutability, weakly normal property, and subnormaliser condition are equivalent for *p*-subgroups of a given group.

**Theorem 4 (see [6], Proposition 3.3).**

Let *H* be a *p*-subgroup of a group *G*. Then the following properties are equivalent:

- *H* is a self-conjugate-permutable subgroup;
- *H* is a weakly normal subgroup;
- *H* satisfies the subnormaliser condition.

In this section, it is shown that self-autopermutable subgroups in nilpotent groups are always characteristic.

**Proposition 5 .**

Let *H* be a subgroup of a group *G*.

- If *H* is self-autopermutable, then *H* is weakly characteristic in *G*.
- If *H* is weakly characteristic, then *H* satisfies the subcharacteriser condition in *G*.

**Proof**

(i) If *H* ^*α*^ ≤ *N*_*G*_(*H*), as *H*⊴*N*_*G*_(*H*), we have *HH* ^*α*^ = *H* ^*α*^ *H*. Applying the condition that *H* is self-autopermutable subgroup of the group *G*, we get *H* ^*α*^ = *H*. By definition, *H* is weakly characteristic.

(ii) Let *K* ≤ *G*, such that *H*⊴*K*. We have *H* ^*α*^ ≤ *K* ^*α*^ = *K* ≤ *N*_*G*_(*H*) for every *α* ∈ *N*_Aut(*G*)_(*K*). Since *H* is weakly characteristic in *G*, we have *H* ^*α*^ = *H*. Thus *α* ∈ *N*_Aut(*G*)_(*H*) and the result is obtained.

The following theorem is one of the main results in this section.

**Theorem 6 .**

Let *H* be a subgroup of a nilpotent finite group *G*. If *H* satisfies the subcharacteriser condition then *H* is characteristic in *G*.

**Proof**

Write *G*≅*P*_1_ × *P*_2_ × ⋯×*P*_*t*_, where *P*_*i*_ is a Sylow *p*_*i*_-subgroup of *G*, for 1 ≤ *i* ≤ *t*. We may also write *H*≅*H*_1_ × *H*_2_ × ⋯×*H*_*t*_, with *H*_*i*_ ≤ *P*_*i*_, 1 ≤ *i* ≤ *t*. Since *H* satisfies the subcharacteriser condition in *G*, one can easily see that *H*_*i*_ satisfies the subcharacteriser condition in *P*_*i*_. Therefore *H*_*i*_⊴*P*_*i*_ implies that *H*_*i*_ is characteristic in *P*_*i*_, which proves the result.

Finally, we show that self-autopermutability, weakly characteristic, and subcharacteriser conditions are equivalent, for every subgroup of a nilpotent group.

**Corollary 7 .**

Let *H* be a subgroup of a finite nilpotent group *G*. Then

- *H* is a self-autopermutable;
- *H* is a weakly characteristic;
- *H* satisfies the subcharacteriser condition in *G*.

**Proof**

The result follows by Proposition 5 and Theorem 6.
